# Cyclic fatigue resistance of VDW.ROTATE and Reciproc Blue nickel-titanium files at root canal temperature

**DOI:** 10.34172/joddd.2020.032

**Published:** 2020-09-21

**Authors:** Ahmet Demirhan Uygun

**Affiliations:** ^1^Department of Endodontics, Faculty of Dentistry, Afyonkarahisar Sağlık Bilimleri University, Afyonkarahisar, Turkey

**Keywords:** Cyclic fatigue, Kinematics, Reciprocation, VDW.ROTATE

## Abstract

**Background.** This study aimed to compare the VDW.ROTATE instruments with the Reciproc Blue instruments in different kinematics in terms of the cyclic fatigue resistance.

**Methods.** Sixty instruments, 40 VDW.ROTATE and 20 Reciproc Blue instruments, were divided into three groups (n=20): VDW.ROTATE was used in both continuous rotation and reciprocation, and Reciproc Blue was used in reciprocation only. The cyclic fatigue resistance test was carried out in an artificial canal (60°, r=3 mm) at an intracanal temperature of 35±2°C until fracture, and the time to fracture was recorded in seconds. The data were analyzed statistically using Kruskal–Wallis and Tamhane’s T2 tests (P<0.05).

**Results.** DAll the reciprocating motion groups resulted in a longer mean duration to failure than the continuous rotation motion group (P<0.05).

**Conclusion.** It was observed that the Reciproc Blue instruments had higher cyclic fatigue resistance than VDW.ROTATE instruments (P<0.05). Recent studies have shown that reciprocal movement increases cyclic fatigue resistance compared to rotational movement. The VDW.ROTATE instrument, which has a similar size, design, and alloy as the Reciproc Blue instrument, can also be used by clinicians in reciprocating motion with endo motors capable of reciprocating in different directions. However, even if the cyclic fatigue resistance increases by using VDW.ROTATE instruments in reciprocation, the cyclic fatigue resistance is lower than Reciproc Blue instruments.

## Introduction

Nickel‒Titanium (NiTi) instruments with superelasticity and shape memory have been preferred by clinicians, especially in the preparation of curved root canals since their first use by Walia et al^1^ in 1988. Despite the increased physical properties of NiTi instruments, instrument fracture in the root canal, albeit infrequent, has been a serious concern for clinicians.^2,3^ Cyclic fatigue, one of the two main instrument fracture mechanisms, results from stress due to successive tension/compression periods at the canal’s most inclined point.^4^

In recent years, manufacturers have developed production techniques that are patented and not fully disclosed to achieve better mechanical properties in NiTi instruments, such as fracture resistance, cutting efficiency, and flexibility. The Reciproc Blue system (VDW, Munich, Germany), a single-instrument system, has a similar morphology to the previous Reciproc system. However, unlike the Reciproc system produced with M-wire technology, the Reciproc Blue system is produced with a new heat treatment technology. As a result, the system has a blue titanium oxide layer on its surface, with increased flexibility and cyclic fatigue resistance.^5,6^ The VDW.ROTATE system (VDW, Munich, Germany), a multi-instrument system, is also manufactured by VDW. This innovative system offers a wide range of instruments for different clinical cases: (15/.04), (20/.05), (25/.04), (25/.06), (30/.04), (35/.04), (40/.04), (50/.04), (60/.04), (30/.06), (35/.06), and (40/.06). Instruments with an S-shaped cross-section are used with clockwise (CW) rotary motion, as indicated by the system name.^7^ The most important difference between these two similar instrument systems produced by the same manufacturer is that one is a reciprocal single-file system, and the other is a rotary multi-file system. In addition, these instrument systems perform cutting by rotating in different directions.

In the first instance, after the glide path was performed, the canal preparation in the reciprocal motion was completed with a single NiTi instrument by Yared.^8^ After this, the reciprocal motion became increasingly popular, and instruments designed to be used in reciprocal movement became commercially available. When researchers compared reciprocal and rotary movements, it was seen that the reciprocal movement had some advantages in some parameters.^9^

Studies that examined the effect of continuous rotation and reciprocal movements with different angles on the cyclic fatigue resistance of instruments have shown that continuous rotation motion significantly decreased the fracture strength of the instruments.^10,11^ On the other hand, NiTi instruments used with rotary motion are still marketed and preferred by clinicians.

There is no evidence that the manufacturer’s selected motion is the best choice concerning cyclic fatigue resistance. Thus, this study aimed to compare the cyclic fatigue resistance of two innovative NiTi instruments, Reciproc Blue R25 and VDW.ROTATE (25/.06), by disabling the effect of motion. The null hypothesis was that there would be no significant difference between the Reciproc Blue and VDW.ROTATE instruments in different kinematics.

## Methods

Twenty Reciproc Blue R25 instruments and 40 VDW.ROTATE instruments with a tip size of #25 and 0.06 taper were used in this study. The instruments were inspected for manufacturing defects using a dental operating stereomicroscope (Novex, Arnhem, Holland) at ×15 magnification before the cyclic fatigue test, and no instruments were excluded from the experiment.

The cyclic fatigue resistance test was carried out in a custom-designed device that simulated an artificial canal made of stainless steel, with a 60° angle of curvature and a 3-mm radius of curvature located 5 mm from the tip of the instrument at 35±2°C in saline solution. The custom-designed device was adjustable in three dimensions and provided the standard position for each instrument. In all the groups, the instruments were operated at 300 rpm with an electric motor and contra-angle handpiece that enabled adjustable rotary and reciprocating kinematics in both CW and counterclockwise (CCW) directions until fracture occurred. The experimental groups in the study were as follows (n=20 for each group):

1. VDW.ROTATE: CW continuous rotary motion was used for instrumentation at 300 rpm.

2. VDW.ROTATE (in reciprocation motion): A reciprocal motion, CW=150° and CCW=30°, was used for instrumentation at 300 rpm.

3. Reciproc Blue: A reciprocal motion, CCW=150° and CW=30°, was used for instrumentation at 300 rpm.

A stopwatch was used to monitor the time to fracture in seconds. The lengths of the detached fragments (LDF) were measured by a digital caliper. The fractured surfaces of the six instruments were evaluated under a scanning electron microscope (LEO 1430 VP, Zeiss Oberkochen, Germany). The Kruskal–Wallis H test was then performed to statistically analyze the time to fracture, and Tamhane’s T2 test was used to determine any statistical significance between the two instruments. The statistical significance level was set at 5% (SPSS v.23.0; IBM Corp, Armonk, NY).

## Results

Significant differences were observed among the groups in terms of the time to fracture (P<0.001) (Table 1). Statistical analysis revealed that Reciproc Blue instruments exhibited significantly better resistance than the VDW.ROTATE groups in the artificial stainless steel canal used in this study (P<0.05). Also, statistical analysis showed that when the VDW.ROTATE instruments were used in reciprocal motion, the fracture resistance increased compared to the rotation motion (P<0.05). The mean of the LDF for Reciproc Blue instruments was significantly higher than that for the VDW.ROTATE groups (P<0.05). There was no significant difference in LDF when VDW.ROTATE instruments were used in rotation or reciprocal motion (P>0.05).

**Table 1:** The mean and standard deviation values for the time to fracture (TTF) in seconds and the length (mm) of the detached fragments (LDF) of the tested instruments

	**TTF**	**LDF**
**VDW.rotate**	134.7±9.7^a^	4.59±0.05^a^
**VDW.rotate (rec)**	384.4±24.1^b^	4.58±0.04^a^
**Reciproc Blue**	407.3±29.3^c^	5.92±0.11^b^

Different superscript letters indicate a statistically significant difference (P<0.05).

## Discussion

This study compared the cyclic fatigue resistance of Reciproc Blue and VDW.ROTATE instruments used in continuous rotation and reciprocation kinematics. The null hypothesis was rejected because there were statistically significant differences between these two instruments, even if the VDW.ROTATE instruments were used in reciprocation.

In the past, most cyclic fatigue resistance studies were performed at room temperature, but this did not reflect clinical conditions, as the root canal had a different temperature. The environmental conditions in which the cyclic fatigue test is performed affect the fracture resistance as well as motion kinematics, metal alloy, and physical properties of the instruments.^12-14^ According to the results of the in vivo studies wherein a limited number of root canal temperature was measured, the root canal temperature was found to range between 31°C and 35°C.^15,16^ Accordingly, in this study, a cyclic fatigue resistance test was performed in saline solution at 35°C (±2°C) using a custom-made temperature control device.

Some previous studies have found a significant difference between the mean LDF.^17-19^ Differences in the designs and alloys of the instruments might change the positions of the maximum stress points. In this study, the mean LDF was significantly different in the Reciproc Blue instruments compared to the VDW.ROTATE instruments, and there was no significant difference in LDF when VDW.ROTATE instruments were used in different kinematics. The low standard deviation of the groups, rather than the absence of difference between the groups, might indicate that standardization is achieved in the cyclic fatigue resistance test.

Previous studies have shown that reciprocal movement prolongs the files’ usage time compared to the rotation movement.^11,20^ Therefore, in a study performed to compare these files with similar physical properties, it was predicted that the VDW.ROTATE instruments used with rotation motion might have lower fracture resistance than Reciproc Blue instruments used with reciprocal motion. To eliminate the effect of motion kinematics, the VDW.ROTATE instruments were tested and compared with the motion of the Reciproc Blue, and the number of groups increased to three.

Reciproc Blue files are recommended for use in a reciprocation mode where the angles of motion and speed cannot be changed in endo motors manufactured by the same company. In the reciprocation mode, the motion and speed angles are CCW = 150° and CW = 30° and 300 rpm, respectively.^21^ However, VDW.ROTATE is recommended for CW continuous rotation at 300-400 rpm.^22^ Since the direction of cutting edges of the instruments is designed differently, one cuts the dentin in CW while the other cuts the dentin in CCW direction. Accordingly, this study used an endo motor capable of reciprocal and rotary motion in clockwise and counterclockwise directions, and in which angles and speed can be adjusted.

Previous studies on endodontic motors have claimed that endo motors do not work under the reciprocation angle values given by their manufacturer. First, Fidler^23^ claimed that the kinematic of reciprocation is more complicated than it seems, as it is described only using angles and rotational speed, and the actual kinematic values of VDW Silver (VDW, Munich, Germany) and ATR Tecnika (Tecnika, Pistoia, Italy) endodontic motors differ from those declared by their manufacturers. Irmak and Ozgur^24^ showed inconsistency between the reciprocal angle values of the endodontic motor X-Smart Plus and the values given by the company. They also showed differences in reciprocal angle values between new and used endodontic motors. In the present study, the Genius Eze endodontic motor was preferred because it can rotate and reciprocate clockwise and counterclockwise at different angles. However, to the best of our knowledge, there was no study examining the accuracy of this motor’s reciprocal motion values. This is the only limitation of the present study. Considering the possibility that the endodontic motor does not work under the actual values, the Reciproc Blue R25 and VDW.ROTATE (25.06) instruments were compared in the same reciprocating mode. Only the direction of the motion was changed. The data obtained in the study were assumed to be accurate, and statistical evaluation was performed.

In this study, reciprocal motion groups exhibited higher fracture resistance than the rotation motion group, consistent with previous studies.^10,11,25,26^ However, previous literature shows that the stress concentrated on the NiTi instruments in the continuous rotation motion at a certain point decreases by reversing the reciprocal motion, and how this difference occurs has not been fully revealed. Gambarini et al^27^ claimed that a full rotation cycle was completed with more reciprocal cycles in the reciprocating movement, and that the reciprocating cycle required more time for one full rotation. Therefore, lower speed and a higher number of reciprocating cycles provide increased cyclic fatigue resistance.

According to the results of this study, the cyclic fatigue resistance of Reciproc Blue instruments was significantly higher than that of VDW.ROTATE instruments, both in rotation and reciprocation kinematics. To the best of our knowledge, as there is no study on VDW.ROTATE in the literature, the current results cannot be compared with those of any other study. The VDW.ROTATE instrument consists of a series of heat-treated, blue instruments, such as the Reciproc Blue instrument.

**Figure 1:**
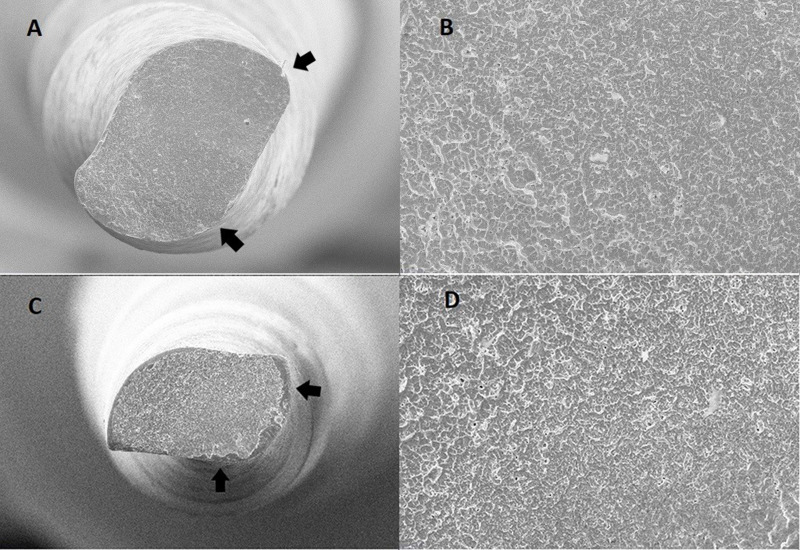
Scanning electron microscopic appearances of the Reciproc Blue and VDW.rotate instruments after cyclic fatigue testing. The fracture surface view of Reciproc Blue (A), VDW.rotate (C), and a high-magnification view of the Reciproc Blue (B) and VDW.rotate (D) instruments. The crack initiation origins (arrows) are observed in the fracture surface.

Moreover, in the scanning electron microscopy images of the fractured surfaces, it has an S-shaped cross-section similar to Reciproc Blue (Figure 1). However, there was a statistical difference between these two instruments, and the findings of this study could be attributed to differences in instrument designs. This must be confirmed by further in vitro investigations.

## Conclusions

Within the limitation of the study, Reciproc Blue instruments exhibited significantly more cyclic fatigue than VDW.ROTATE instruments. When using the VDW.ROTATE instruments with the reciprocal motion, the resistance to fracture increased compared to the continuous rotation motion. This result is not sufficient to endorse the safe use of the VDW.ROTATE instrument (#25,0.6) alone without previous instruments of its own system in reciprocal motion. Further studies are needed to examine parameters such as torsional fatigue, cutting efficiency, and debris removal. After such studies, the clinical use of the VDW.ROTATE instrument in reciprocal motion might be of interest.

## Authors’ Contributions

All stages of the present study were undertaken and carried out by the author.

## Acknowledgments

None.

## Funding

The study has not been supported by any organization.

## Competing Interests

The author declares no conflicts of interest.

## Ethics Approval

This article does not contain any studies with human participants or animals performed by the author.
